# DNA Aptamers for the Functionalisation of DNA Origami Nanostructures

**DOI:** 10.3390/genes9120571

**Published:** 2018-11-23

**Authors:** Yusuke Sakai, Md. Sirajul Islam, Martyna Adamiak, Simon Chi-Chin Shiu, Julian Alexander Tanner, Jonathan Gardiner Heddle

**Affiliations:** 1 Malopolska Centre of Biotechnology, Jagiellonian University, 30-387 Krakow, Poland; yusuke.sakai@uj.edu.pl (Y.S.); md.sirajul.islam@uj.edu.pl (M.S.I.); martyna.adamiak@uj.edu.pl (M.A.); 2 School of Biomedical Sciences, Li Ka Shing Faculty of Medicine, The University of Hong Kong, Hong Kong, China; simon.chichin.shiu@gmail.com (S.C.-C.S.); jatanner@hku.hk (J.A.T.)

**Keywords:** DNA origami, aptamer, DNA nanotechnology, protein nano array, biosensor, logic gate, enzyme cascade, drug delivery system, targeted therapy, molecular robotics

## Abstract

DNA origami has emerged in recent years as a powerful technique for designing and building 2D and 3D nanostructures. While the breadth of structures that have been produced is impressive, one of the remaining challenges, especially for DNA origami structures that are intended to carry out useful biomedical tasks in vivo, is to endow them with the ability to detect and respond to molecules of interest. Target molecules may be disease indicators or cell surface receptors, and the responses may include conformational changes leading to the release of therapeutically relevant cargo. Nucleic acid aptamers are ideally suited to this task and are beginning to be used in DNA origami designs. In this review, we consider examples of uses of DNA aptamers in DNA origami structures and summarise what is currently understood regarding aptamer-origami integration. We review three major roles for aptamers in such applications: protein immobilisation, triggering of structural transformation, and cell targeting. Finally, we consider future perspectives for DNA aptamer integration with DNA origami.

## 1. Introduction

**Figure 1:**
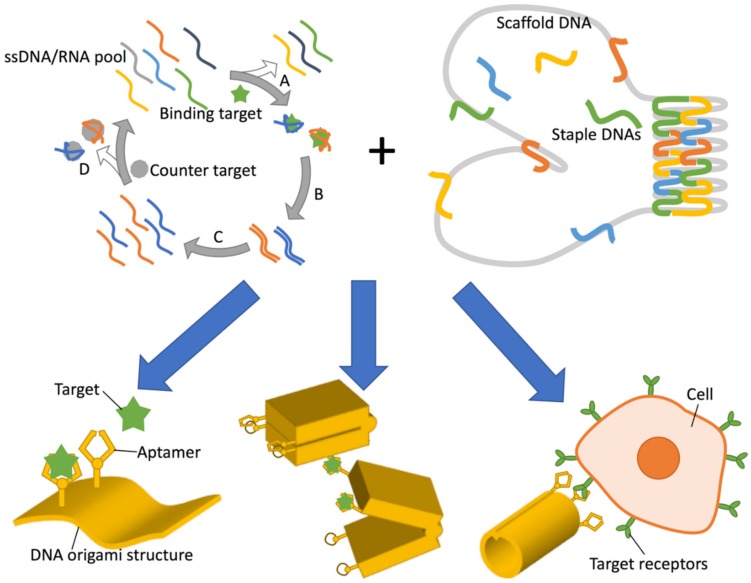
Applications of DNA aptamers when combined with DNA origami. (**Top left**) Schematic of the SELEX (Systematic Evolution of Ligands by EXponential enrichment) process. First, a fraction with target (green star) affinity is extracted from an initial ssDNA (single-stranded DNA)/RNA pool (**A**) and amplified by (reverse transcription and) PCR amplification (**B**). The reverse sequences are removed or forward sequences are transcribed to prepare an enriched ssDNA or RNA library, respectively (**C**) and occasionally counter selection is inserted to remove non-specific binding fractions according to affinity for the counter target (grey circle) (**D**). The selected fraction is used as the second-generation library for the next round of selection (**A**). (**Top right**) A schematic illustration of DNA origami assembly. A long ssDNA (grey) is folded by hundreds of short ssDNA (coloured) by designed hybridization. **Bottom**: Combination of aptamer and DNA origami (yellow) can result in: (**Bottom left**) protein nanoarray and biosensor construction via capture of target protein (green) by the aptamer arm; (**Bottom center**) Target dependent mechanical transformation by aptamer-lock or split aptamer integration for either biosensing or molecular computing; and (**Bottom right**) specific cell targeting for drug delivery and targeted therapy e.g., by utilising cancer cell targeting aptamers.

Nucleic acid aptamers are typically 15–90-nucleotide lengths of single-stranded DNA, RNA, or modified nucleic acid which can function similarly to antibodies—having the ability to bind to molecular targets with high specificity and affinity. They are selected from a random sequence pool according to affinity for a particular target by several rounds of selection and amplification in a process known as Systematic Evolution of Ligands by EXponential enrichment (SELEX) (Figure 1) [1,2]. This was originally carried out using RNA, however single-stranded DNA (ssDNA) was shown to be viable in work that developed a thrombin aptamer [3]. The use of DNA has a number of advantages over RNA, notably higher chemical stability and obviation of a reverse transcription step during each round of SELEX, and concomitantly, DNA aptamer development has grown rapidly [4]. Perhaps surprisingly, although RNA has the additional 2′-hydroxyl group which imparts more diverse secondary structures than DNA, successful aptamers of both types have been shown to have affinities comparable to monoclonal antibodies (*K*_D_ in the range 0.1–50 nM [4]). Aptamers are synthesised chemically, allowing a variety of chemical modifications to be introduced to the base, ribose, and phosphate backbone, thereby conferring desirable properties such as increased chemical stability, attachment points for further chemical conjugation, and higher affinities for targets [5]. For example, 2′-fluoro, 2′-amino and 2′-*O*-alkyl substitution of ribose are well-known to increase nuclease stability [6,7], while introducing amino acid mimicking bases has been demonstrated to expand the chemical diversity of aptamer structures [8,9].

As for antibodies, aptamers have the potential for targeted therapy, triggering cell signalling, or the inhibition of the enzymatic activity of target molecules. Unlike the lengthy, laborious, and expensive production of antibodies, aptamers can be readily chemically synthesized which is cost efficient and reduces the costs for medical usage. Further advantageous characteristics of nucleic acid aptamers include flexibility of chemical modification, long storage period, and low immunogenicity. Conjugation to other chemical entities has been demonstrated, including to chemotherapeutic agents, siRNA, various nanoparticles, and onto solid phase surfaces for therapeutic and diagnostic applications [5]. A notable example of practical use is Macugen, an RNA aptamer that was the first Food and Drug Administration (FDA) approved drug that was used in the treatment of macular degeneration [10,11]. Several aptamer therapeutics have shown promise in pre-clinical models and are progressing in oncology clinical trials [12]. For clinical usage, it is a challenge to protect the aptamer from enzymatic degradation. Introducing modified nucleic acids and coating with polyethylene glycol (PEGylation) are common approaches to extend circulation turnover [13]. Aptamers are also being developed that can be used as clot busters, cancer therapies, autoantibodies, diabetes treatments, etc. [14].

Scaffolded DNA origami (also herein occasionally referred to as simply “DNA origami” or “origami”) is a method whereby arbitrary 2D and 3D nanoscale objects made from DNA can be designed and produced in a relatively straightforward way (Figure 1) and was first introduced in 2006 [15]. DNA origami structures are made from a scaffold strand of ssDNA, typically M13 phage genomic DNA which is folded into shape by many (typically around 200) short staple strands which are complementary to linearly distal sequences of the scaffold strand. Maximisation of base-paring to the staple strands brings the distal sequences into close proximity and in this way, a targeted shape can be formed. DNA origami has progressed rapidly and a breathtaking array of structures have been produced [16]. 2D DNA origamis have been widely used, for example, to produce nanoscale imagery [15,17] as well as structures with potential applications such as molecular pegboards [18,19,20,21] and molecular rulers [22,23] and to produce heterogenous arrays of proteins in nanoscale wells [24]. Lattice-based 3D DNA origamis [25] have been produced with a wide range of sizes, shapes, and decorations. These have been shown to be able to act as nanocontainers [26], immunomodulating agents [27], computational devices and standards in electron microscopy [28], amongst many others. Wireframe-based DNA origami offers both 3D and 2D structures with more diverse geometries [29,30,31]. As these hollow structures consist of single helix or bundled double helices edges connected by multi-arm junctions, DNA helices are less packed than lattice-based 3D DNA origami in nature and are more stable under physiological salt conditions, which is more suitable for biomedical applications [16].

As DNA origami and DNA/RNA aptamers are constructed from nucleic acid material, they offer an immediate compatibility with DNA origami whereby aptamers may be attached to any extended staple sequence via base pairing or via simple extension of the staple sequence. Aptamers can potentially be placed anywhere on a DNA origami structure with high resolution due to the fact that the DNA helices in DNA origami structures are bundled tightly together with the centres of neighbouring helices being only ca 2 nm apart and the phase of the double helical phosphate backbone of the staple stands to which the aptamers attach repeats with a ca 3 nm pitch. It is also the case that each staple strand could in theory be appended with a different bound molecule, allowing for multiplexing of functionalities. Furthermore, existing DNA origami structures held together by strand-displacement locks [26,32,33,34] have provided a convenient start-point whereby the locks could be replaced with aptamer sequences to provide ligand-binding based unlocking mechanisms [26].

DNA aptamers have found a myriad of uses on nanometric structures which themselves are constructed of materials other than DNA [35]. For example, they have been attached to gold nanoparticles whereby the presence/absence of ligands such as lead, adenosine, cocaine, or mixtures can control the accessibility of complementary sequences on the nanoparticles leading to “switching on” or “off”, resulting in observable colour changes [36,37]. Aptamers responsive to ATP have been used to decorate polymers to deliver doxorubicin, an anti-cancer drug, designed to be released in the cell due to ATP aptamer transformation [38,39].

DNA origami research is a part of a vast field of DNA nanotechnology within which there are numerous reports of aptamer functionalised DNA nanostructures [40,41,42,43,44,45]. Though still at a relatively early stage, three principles of successful aptamer integration design for DNA nanostructures have recently been proposed, namely (i) Shape—taking into account the overall DNA nanostructure design including the location and expected function of aptamers; (ii) Self-Complementarity—the degree of self-complementarity of an aptamer lock to itself and/or partner strand is important for controlling equilibration between the two states of the aptamer in the presence or absence of the target molecule; (iii) Spatial Flexibility—aptamers need to be positioned on DNA origami structures so that they do not interfere with the desired function such as conformational changes [46]. As more aptamers are developed, they will represent a growing library of motifs that may be incorporated into DNA origami structures. Here, we highlight specific examples of DNA origami research as well as milestone reports of individual methodologies to give a snapshot of the current research and future perspectives of the marriage between DNA origami and nucleic acid aptamers.

**Figure 2:**
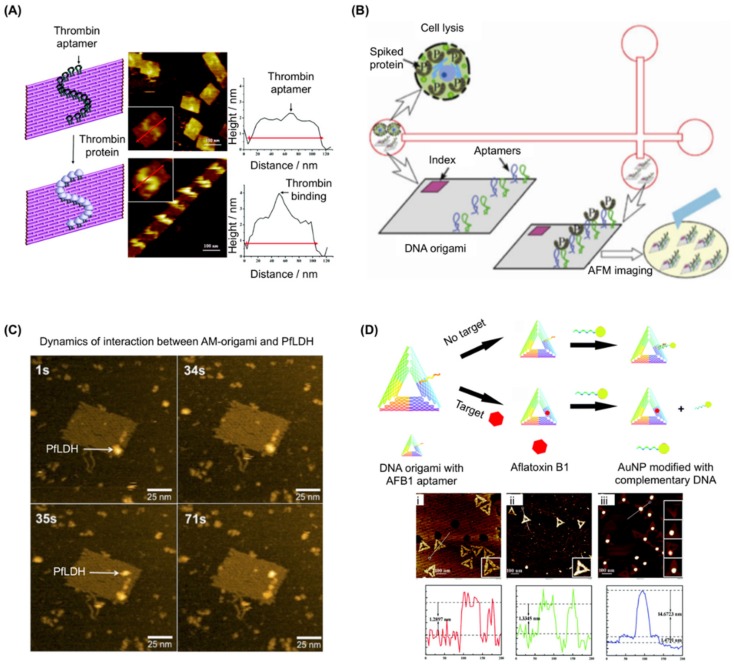
Examples of aptamer use in target immobilization on DNA origami. (**A**) (**left**) Schematic showing an S-shaped pattern of thrombin templated on DNA origami. Grey balls represent thrombin proteins. (**middle**) Images corresponding to the arrays shown on the left, with enlarged images (inset); (**right**) line cross-section analysis of the atomic force microscopy (AFM) images show an increase in the height at the sites of protein binding. Adapted with permission from Chhabra et al. [18]. Copyright (2007) American Chemical Society. (**B**) Schematic for the stacking, separation, and identification of DNA origami and its protein binding using isotachophoresis (ITP) in a cross-channel microfluidic chip fabricated in fused silica. DNA origami with bivalent aptamers was assembled by replacing individual staple strands. Thrombin was used as the target analyte to provide functional validation. After incubating origami in thrombin-spiked cell lysate, separation was performed by on-chip ITP and was verified by direct visualization with AFM. Adapted with permission from Mei et al., Nano Res., 2013 [54]. (**C**) High speed (HS)-AFM images showing the dynamics of the interaction between AM-origami and *Plasmodium falciparum* lactate dehydrogenase (PfLDH). At 1 s, a single AM-origami is visible with one PfLDH (indicated by a white arrow) attached to the aptamer-modified region. At 35 s, a second PfLDH (indicated by a white arrow) binds to the region and both proteins remain in place beyond 71 s. Adapted from Godonoga et al. [57]. (**D**) (**top**) Schematic illustration of the analytical principle of the aptamer-tagged DNA origami/complementary ssDNA-AuNPs system for detecting aflatoxin B1 (AFB1); (**bottom**) AFM images with section plots of aptamer-tagged triangular DNA origami nanostructures before and after binding. (i) Aptamer-tagged triangular DNA origami; (ii) aptamer-tagged triangular DNA origami after binding with AFB1; (iii) aptamer-tagged triangular DNA origami after hybridization with the AuNP-conjugated ssDNA. From Lu et al., Chem. Commun., 2017 [58]—Reproduced by the permission of The Royal Society of Chemistry. AuNP: Gold nanoparticles.

**Figure 3:**
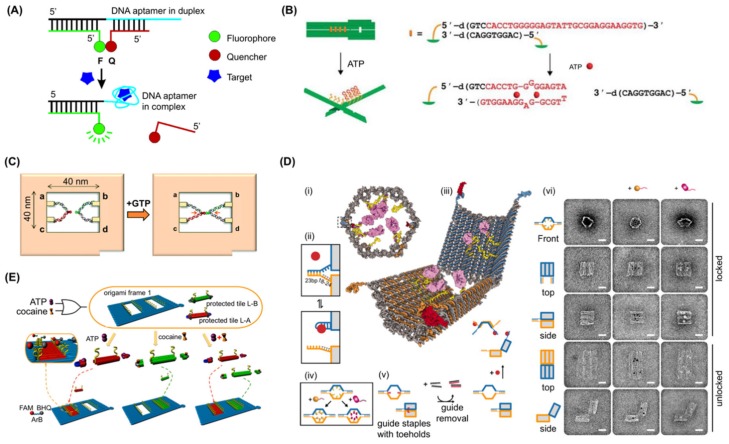
Examples of aptamer use in controlling DNA origami structural change. (**A**) Schematic for designing aptamer-based fluorescent reporters that function by switching structures from DNA/DNA duplex to DNA/target complex. Adapted with permission from Nutiu et al. [98]. Copyright (2003) American Chemical Society. (**B**) Key and lock system to detect ATP using aptamer-based elements. Adapted from Kuzuya et al. [78]. (**C**) Schematic of the system for investigating the interaction of kissing RNA aptamers using a DNA frame. Incorporation of the aptakiss into the a–c site and KG51 or GTPswitch into the b–d site in the DNA frame. When the GTPswitch is incorporated into the DNA frame, GTP should induce conformational change from the double-loop to the X-shape. From Takeuchi et al. [85]—Reproduced by the permission of The Royal Society of Chemistry. (**D**) (**i**) Schematic of the front view of a closed nanorobot loaded with a protein payload; (**ii**) the DNA aptamer (blue) and a partially complementary strand (orange) make the aptamer lock. The lock can be stabilized in a dissociated state by its antigen key (red); (**iii**) nanorobot opened by protein displacement of aptamer locks. The two origami domains (blue and orange) are constrained in the rear by scaffold hinges; (**iv**) Payloads (gold) and antibody Fab’ fragments (magenta) can be loaded inside the nanorobot; (**v**) Front and side views show guide staples (red) bearing 8-base toeholds that aid the assembly of the nanorobot. After folding, guide staples are removed by the addition of fully complementary oligos (black). Nanorobots can be subsequently activated by interaction with antigen keys (red); (**vi**) TEM images of robots in closed and open conformations. From Douglas et al. [70]. Reprinted with permission from the American Association for the Advancement of Science. (**E**) Schematic illustration of an “OR” gate using ATP and cocaine as two independent inputs to trigger the filling patterns. In the locking step, both tile A or B can be deactivated by hybridizing with the protector strands PA or PB, which contain aptamer-recognizing sequences of ATP and cocaine in the middle region, respectively. In the unlocking step, the protector strands (PA or PB) at the ends of the L-A or L-B can be removed by adding the aptamer target (ATP or cocaine). Adapted with permission from Yang et al. [79]. Copyright (2016) American Chemical Society.

**Figure 4:**
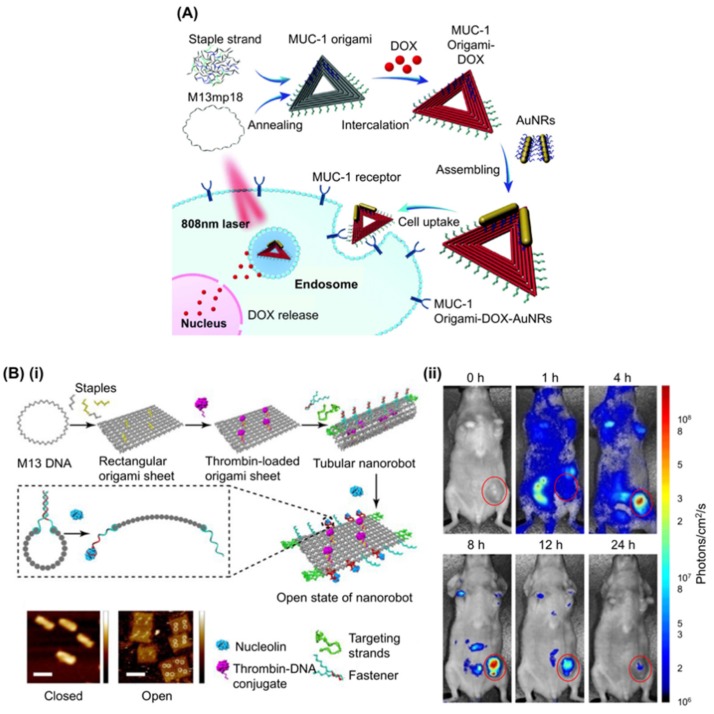
Examples of aptamer use in cell targeting of DNA origami. (**A**) Schematic showing that triangle DNA origami functionalized with the Mucin 1 protein (MUC-1) aptamer can load doxorubicin and carry AuNRs to inhibit the growth of resistant breast cancer cells. The triangular shaped DNA origami structure is hybridized with staple strands (grey), MUC-1 aptamer strands (green), and capture strands (blue). The multifunctional DNA nanostructures (MUC-1 aptamer–DNA origami–DOX–AuNRs complex, MODA) were administered to the MCF-7/ADR cells and the photothermal effects were investigated. Adapted with permission from Song et al., Nanoscale, 2017 [87]. (**B**) (**i**) (**top**) Schematic showing the construction of a thrombin-loaded nanorobot by DNA origami and its change of shape into a rectangular DNA sheet in response to nucleolin binding. (**bottom**) AFM images of the DNA nanorobots in closed (**left**) and opened states (**right**). The four bright spots displayed on the surface of the origami sheet represent the thrombin molecules. (**ii**) In vivo experiment to show the activity of aptamer-based DNA nanorobots. Optical imaging of an MDA-MB-231 mouse bearing a human breast tumor before and after intravenous injection of Cy5.5-labeled nanorobot. A high-intensity fluorescent signal was detected only in the tumor region of mice 8 h after injection. 0 h indicates before injection. Adapted with permission from Li et al., Nat. Biotechnol., 2018 [91]. AuNR: Gold nanorod; DOX: doxorubicin.

Overall, DNA and RNA aptamers offer significant opportunity for facile attachment to designed DNA origami structures in order to endow them with useful functionality. Existing research points at the possible categories of aptamer modifications that may be utilised and these include (i) aptamers to immobilise target molecules, as demonstrated by nanoarrays, as well as biosensor applications (Figure 2); (ii) using aptamers to trigger conformational changes of DNA nanostructure aimed at either biosensor or molecular computing outcomes (Figure 3); (iii) using aptamers for (cancer) cell targeting for drug delivery (Figure 4).

## 2. Aptamers for Target Immobilization

DNA nanotechnology offers an exquisite method for producing bottom-up nanoarrays, i.e., it can be used to organize various particles in spatially defined patterns with nano-meter precision via self-assembly, as first demonstrated by the construction of simple nanostructures of gold nanoparticles precisely arrayed on a DNA helix [47]. Using biotinylated DNA has allowed templated nanoarrays to expand to include streptavidin [48] and streptavidin-coupled antibodies [49]. A more universal methodology of DNA-based protein nanoarray formation was subsequently shown [50] where a 15nt thrombin aptamer (HD1) [3] was successfully introduced which precisely arrayed thrombin on triple-crossover DNA tiles as observed by atomic force microscopy (AFM). These arrayed nanoparticles are typically visualised by AFM as dot(s) of appropriate height on regularly patterned DNA nanostructures as if pegged by aptamers on pegboard made of DNA.

In 2007, just after the introduction of the DNA origami method, the platform of aptamer-based protein nano-arrays was expanded from DNA tiles [50] to DNA origami (Figure 2A) [18]. A platelet derived growth factor (PDGF) aptamer (36t) [51] and a thrombin aptamer (HD22) [52] were each arrayed on a rectangular DNA origami structure with nanometre precision. This multivalent aptamer system was further systematically investigated [53]: one target molecule was captured on the DNA nanostructure by two aptamers recognising different epitopes using another thrombin aptamer (HD1) [3]. Utilising the precise addressability inherent to DNA nanostructures, 4-helix bundle or 5-helix bundle DNA tiles and rectangular DNA origami were arrayed with two thrombin aptamers with a separation varying from 2 to 6.9 nm in order to seek the optimal interval for the efficient binding of thrombin, verified by electrophoretic mobility shift assay (EMSA). A four-thymine spacer was added to the ends of the aptamers to increase flexibility. As the two aptamers recognise opposite sides of the ~4.1 diameter protein simultaneously, the optimal spacing of the aptamers was found to be 5.3 nm, i.e., four double-stranded DNA (dsDNA) helices. Combining a microfluidics system and the DNA origami with dual thrombin aptamers resulted in a biosensor system for the rapid detection of thrombin from cell lysate (Figure 2B) [54]. In this work, a simple crossed microchip for isotachophoresis (ITP) was prepared, where a mixture of DNA origami and thrombin spiked cell lysate was electrophoresed and concentrated between the loading electrolyte (LE) and the trailing electrolyte (TE). The concentrated fraction of the DNA origami-thrombin complex was then extracted from the cross section of the microfluidics chip and was confirmed by AFM. Successful thrombin detection from cell lysate spiked when 15 nM thrombin was achieved. Recently, all atom molecular dynamics studies were performed to simulate the binding behaviour of a dual thrombin aptamer system with a 1152nt scaffold small DNA origami structure, 26nt thrombin aptamer (NU172, also known as ARC2172) [55], and 29nt-long thrombin aptamer (HD22) [56].

Rothemund’s rectangular DNA origami structure was utilised by several groups as a basis for pegboard biosensor construction. For example, dual thrombin aptamers (HD1 and HD22), inactivated by a guanine base O^6^-methyl modification in advance, have been used to detect the activity of the DNA repair enzyme, human O^6^-alkylguanine-DNA alkyltransferase (hAGT) [59]. When a sample mixed with the biosensor has demethylation activity, the original sequence of the thrombin aptamer is recovered and will immobilise thrombin on DNA origami, which is visible in AFM. As the inhibition of hAGT can enhance the cytotoxicity of alkylating agents in tumour cells, this system has a potential use in screening inhibitors as candidate chemotherapy enhancers. In another example [57] we developed a DNA origami biosensor with potential for malaria detection by integrating recently discovered *Plasmodium falciparum* lactate dehydrogenase (PfLDH) aptamers (2008s) (Figure 2C) [60]. This is notable as PfLDH is a biomarker of malaria infection. Twelve PfLDH aptamers were arrayed on a DNA rectangle via 20nt poly T linkers and specific binding of PfLDH was observed using AFM and high-speed AFM (HS-AFM) measurements. Although the affinity was decreased when the aptamer was connected to staple strands (Native *K*_D_ ≈ 56 ± 18 nM, staple connected: *K*_D_ ≈ 600–1100 nM), the aptamer modified DNA rectangle selectively captured PfLDH at concentrations as low as 500 nM and functioned even in the presence of blood plasma. Captured PfLDH retained enzymatic activity allowing biochemical detection using methods other than AFM imaging. Others have constructed a DNAzyme-operated logic gate system that is able to release a detectable analyte only in the presence of certain DNA strands [61]. In these conditions, a DNA sequence on the DNA origami surface was cleaved by a DNAzyme, leading to ssDNA release. The released DNA could be pre-labelled with detectable moieties such as ruthenium dye and gold nanoparticles (AuNP). Protein capture and programmed release was demonstrated by incorporating the thrombin aptamer (HD1) into the cleavable strand [61] with released thrombin being detectable e.g., via SDS PAGE.

In other work, a NOT gate was constructed on a triangular DNA origami structure to detect the small molecule, aflatoxin B1 (AFB1), using the pegboard-based detection method (Figure 2D) [58]. Here, staple strands were modified with an AFB1-binding aptamer [62] sequence. In the absence of AFB1, a complementary sequence with an AuNP attached was able to hybridise to the aptamer, detectable via AFM and gel electrophoresis. If AFB1 is present, it is bound by the aptamer meaning that the gold-labelled strand cannot bind. This NOT gate system enables the detection of a small molecule target that is not visible in AFM. Other alternatives to microscope imaging-based methods for detecting binding to aptamers on origami have been demonstrated with one example using surface plasmon resonance to evaluate the interaction of a 3D DNA origami structure with thrombin aptamer (HD22) and thrombin [63].

Since aptamers can precisely localise proteins onto the surface of DNA origami, we foresee that this will be a useful approach for biophysical characterisation of enzymes and for nanoreactors. Similar ideas have been demonstrated using protein-DNA conjugates on various designs of origami. For example, glucose peroxidase and horseradish peroxidase have been physically coupled through origami for the peroxidation of 2,2′-azino-bis[3-ethylbenzothiazoline-6-sulfonate] (ABTS) or 3,3′,5,5′-tetramethylbenzidine (TMB), resulting in signal generation, as reviewed in 2017 [64]. Since DNA origami is highly programmable, the functionalisation of this enzyme cascade could be optimized through varying the spacing distance between enzymes, thereby providing insight into the mechanism [65].

As aptamers can be defined as single-stranded nucleic acids binding specifically to a target, known sequences which bind to a specific protein could also be regarded as naturally occurring analogues of aptamers. The Morii group has developed Zinc-finger [66] or leucine-zipper [67] based DNA binding motifs to array target proteins on DNA nanostructures. In 2016, they created an artificial enzyme cascade by incorporating binding sites for xylose reductase and xylitol dehydrogenase at specific locations on DNA origami [68]. Both binding sites were single stranded DNA and one of them had a benzylguanine modification on thymine for binding to xylitol dehydrogenase. This design of origami resembled the xylose metabolic pathway. Enzymatic activity resulting in the generation of xylulose and NADH could also be controlled through modulating inter-enzyme distances. Recent work from the same group also demonstrated the use of zinc-finger protein adaptors consisting of single-stranded hairpin structures for the binding of Kir3 K^+^ channel proteins [69]. The precise fabrication allowed the design of various cavities in the DNA origami such that the K^+^ channel current activity could be controlled by the oligomerisation state of the protein complex. The result demonstrated the potential of aptamer-functionalised DNA origami to modulate membrane protein activity.

In summary, DNA origami has expanded the concept of protein nanoarrays which was formerly investigated only on relatively smaller DNA nanostructures. Combining AFM visualisation and simple logic gates, a series of biosensor devices have been proposed targeting thrombin, PDGF, hAGT activity, PfLDH, and AFB1. Challenges include the fact that the integration of aptamers into DNA nanostructures may suppress the affinity for the target due, for example, to the sequence extensions that are required for connection to the larger structure, imperfect DNA origami assembly, and steric hindrance for target molecule which can be partially recovered by optimising aptamer positioning and linker length. Combining simple logic gates with aptamers enables the detection of aptamers targeting particles which may be too small to be easily detectable in AFM imaging. A summary of aptamers used in conjunction with DNA origami are given in Table 1.

## 3. Aptamers for Controlling DNA Origami Structural Changes

Molecular recognition by aptamers is accompanied by conformational change. By optimising the equilibrium between the partially complementary strand hybridisation and target-aptamer binding, aptamers behave as molecular switches, converting the detection of specific target molecules into structural changes of DNA, which can be further converted to detectable outputs such as fluorescence signal shifts (Figure 3A) [98]. If such aptamer modules are included in DNA nanostructures, aptamer-target interaction can be made such that it results in the release of the strand complementary to the aptamer strand, leading to the dynamic transformation of the overall structure (Figure 3B). Where aptamers already consist of two moieties [86] or have been split by design [99], they can be reconstituted into a single complex in the presence of the target molecule. When each moiety of such an aptamer module is integrated to a distal site on the DNA nanostructure, molecular recognition brings the two parts together. In this way, aptamers can be used to actuate motion in DNA origami systems in response to specific molecular signals and different aptamers in parallel can be used to instantiate logic gates. To date, a number of DNA origami structures have demonstrated the use of aptamers in this way as described below.

### 3.1. DNA Origamis with Potential as Biosensors

A biosensor oriented nanomechanical DNA origami structure was initially reported in 2011 (Figure 3B) [78]. This design consisted of a pliers-shaped DNA origami nanostructure of two DNA origami bars covalently connected at their two centres by single crossovers and one to four pairs of sensory modules including ATP aptamers [81,82]. The aptamer and its complementary strands formed four pairs of “aptamer locks” to convert the DNA nano-pliers from an open X-shape to a closed parallel bar shape. In the presence of 1 mM ATP, mimicking cellular concentrations, the aptamer module captures ATP, releasing the complementary strand, resulting in a decrease of the fraction of closed form DNA pliers from 72% to 40%. Target specificity was confirmed using GTP as a negative control. One to four pairs of telomere elements were also integrated, and these were dimerized and formed a G-quadruplex in the presence of sodium (or potassium) ions. The modules acted as a zipper to close the DNA nanostructure from X form to parallel form in the presence of sodium or potassium ions. Thus, the dynamic DNA origami structure converted the binding of small ligand to target aptamer mechanical transformation (opening or closing) of a large DNA origami measurable by AFM imaging at a single molecular level or via a real-time fluorescence spectrum shift of fluorescence resonance energy transfer (FRET) in bulk.

A mechanochemical DNA origami device utilising a PDGF aptamer was demonstrated in 2014 [71]. In this approach, a 7-tile DNA origami nanostructure was designed in which the recognition elements interlock adjacent tiles. The binding of a target to any of the recognition elements releases the lock, which generates a change in the mechanical force signal, constantly measured by optical tweezers. A PDGF aptamer [51] was used as the first recognition element in each of the six interlocks. The detection sensitivity of the 7-tile origami device was as low as 10 pM within 10 min, improving from 100 pM within 30 min for a former single-interlock system [100], implying that the arrangement of multiple sensor units in series enhanced both the detection limit as well as detection time. A multiplexing capacity of the mechanochemical sensing platform was also demonstrated. In the system, a DNA origami was designed with multiple recognition elements. It integrated a pair of DNA hybridisation locks with toehold sequence, which is complementary to target DNA, in addition to the PDGF aptamer for multiplex sensing of the target DNA and the PDGF at the same time [32]. DNA origami and aptamers have been combined to observe RNA kissing complex formation with single molecule resolution [85]. To do this, a frame-shaped DNA origami structure was designed with the two RNA sequences on opposite sides of a central hole. One sequence was a designed aptamer motif (GTPswitch) which binds to the other moiety (aptakiss) responding to the presence of GTP [86]. Before the addition of GTP, both aptamers were visible in AFM as discrete structures, forming a double loop shape. This became an X-shape after the addition of GTP (Figure 3C) [85]. The GTPswitch sequence was optimised to attain a statistically significant detection of 1 mM GTP (65.2 ± 0.5% from 44.0 ± 2.0% in absence of target molecule) and discrimination against ATP (46.4 ± 2.5% X-shape in presence of 1 mM ATP) meaning that GTP detection at a single molecule level was achieved.

As DNA origami is a modular methodology, many groups have introduced various aptamers into established structures for further functionalisation. For example, a split aptamer that was able to bind to two molecules of ATP [84] was introduced into the nanomechanical DNA origami pliers [83]. The aptamers were labelled with different dyes such that a FRET fluorescence spectrum shift would occur if the dyes approached each other (i.e., if the pliers closed). In the presence of 0.1 mM to 1 mM ATP, the split aptamers bound to ATP, reconstituting the native aptamer and closing the DNA pliers, resulting in the successful detection of ATP by FRET in real-time, confirmed by AFM and agarose gel electrophoresis.

Aptamers may have a use in targeting DNA origamis to cancer cells and a set of aptamers for cancer-specific Mucin 1 protein (MUC-1) [89] have been integrated into a spherical DNA origami structure [101]. In the system, two hemispheres are connected by single crossover and five pairs of ssDNA evenly protrude from equatorial helices of both hemispheres. After assembling the opened structure, each pair of the ssDNA overhangs are interlocked by lock strands containing a centrally positioned MUC-1 aptamer sequence. In the presence of the “key”, i.e., the target protein or the complementary strand for the lock strands, the lock strands are removed from the DNA nanostructure, leading to the opening of the sphere. The device was demonstrated to open on exposure to MUC-1-containing cell lysate [88]. Other aptamers used in relation to disease treatment or detection include the PfLDH aptamer [60] which has been shown to work as part of a malaria biosensor/protype therapeutic delivery system whereby the aptamers control the opening of a DNA origami box [77]. In this work, two pairs of aptamer lock with partially complementary strands were integrated between the lid and the main box. The conformational change of the PfLDH aptamer on binding PfLDH competes with the duplex formation closing the box lid. In the absence of the target molecule or the presence of negative control (human LDH-B), the boxes with aptamer locks showed mostly closed conformation (20% of DNA box open according to TEM imaging), while in presence of 100 nM PfLDH, DNA box in the open form reached ca 70% during 120 min of incubation. The opening of the box by FRET based kinetics was also monitored. Due to the complementary strands locking the lid, the estimated *K*_D_ of the aptamer was 655 nM, which was weaker than native aptamer with *K*_D_ of approximately 42 nM.

Other robotic DNA origami devices operated by aptamer “logic gates” can be used as biosensors as demonstrated by Douglas et al. [70], however they are considered in more detail in the following section.

### 3.2. DNA Origami for Molecular Computing

The production of programmable DNA origami machines is a major goal. Indeed, many of the aptamer-operated origami structures to date respond only if aptamer binds to signal and so could be said to be a form of Boolean logic gate. However, beyond the largely sensory modules described above, aptamer integration has offered more sophisticated programmability to DNA origami structures [38,39,102].

One of the most innovative examples was demonstrated in 2012 [70]. Here, a capsule-shaped structure with two pairs of different aptamer locks closing the shape was designed (Figure 3D) and a PDGF aptamer (41t) [51], protein kinase 7 aptamer (Sgc8c) [75,76], and CCRF-CEM cell targeting aptamer (TE17) [74] were incorporated. A cargo of up to 12 antibody (fragments) were encapsulated to target the produced nanorobot to specific cells displaying antigen receptors, enabling it to discriminate cell types amongst Burkitt’s lymphoma, acute myeloblastic leukemia, aggressive NK leukemia, T-cell leukemia, acute lymphoblastic leukemia, and neuroblastoma. Cells not expressing the molecular “keys” to open the aptamer locks were not bound by the loaded DNA origamis, while cells expressing the keys were due to the opening of the structure and exposure of the antibody fragments. By using two different aptamer locks on a single DNA origami structure, an AND gate could be constructed whereby the capsule would open only in the presence of both molecular keys. Due to fluorescence labelling of the antibody fragments, the binding of DNA origami to target cell types could be verified by fluorescence-activated cell sorting FACS [70]. It is interesting to note that a high yield of closed DNA origami structures was obtained by using additional “guide” staple strands to help set the closed state. These could subsequently be removed prior to interaction with the target. In this way, a yield of 97.5% of closed conformation was achieved.

The logic gate concept was subsequently expanded to include more sophisticated programmability ex vivo as well as in vivo using a cockroach model [72]. A barrel-shaped DNA origami structure similar to that described above was designed and PDGF aptamer (41t) and vascular endothelial growth factor (VEGF) aptamer (SE12) [73] locks were used to produce an effector robot (E) with an AND gate. Then, a positive regulator (P) DNA origami was constructed, which was loaded with ssDNA complementary to one of the locking strands of the effector such that binding of the two robots opens E regardless of aptamer-ligand interaction. Similarly, a negative regulator (N) was constructed carrying two ssDNAs complementary to two locks on opposite sides of E, keeping it closed regardless of aptamer-ligand interaction. With a different toehold sequence present on the locking strands, a secondary effector robot (F), which is sensitive to PDGF and VEGF however is not activated or inactivated by positive or negative regulators, was also introduced. Combining P, N, and F robots in addition to the original nanorobot with an AND gate behaviour resulted in the successful emulation of AND, OR, XOR, NAND, NOT, CNOT, and half adder logic-gates.

Other logic-gate systems have been implemented in DNA origami to detect molecules of interest. For example, a DNA origami frame was produced containing two holes which could be filled by DNA tiles in the presence of predefined target molecules, in this case ATP and cocaine (Figure 3E) [79]. The DNA tile modules are inactivated by aptamer-locks [90,97] which prevent them from filling the holes, however in the presence of ATP or cocaine, the aptamer locks are released from the DNA tiles, activating sticky ends that have complementarity to sequences lining the holes. Whether the holes are unfilled or filled can be detected by AFM. In this system, the design elegantly included a mechanism whereby detection could be achieved without requiring AFM. To do this, a DNAzyme (Mg^2+^-dependent E6-type DNAzyme-1) was reconstituted when the tiles were bound into the holes. The reconstituted DNAzyme cleaved a fluorescent reporter which included a fluorophore and quencher such that active DNAzyme resulted in an increase in fluorescent signal. Combinations of these reporter systems enabled emulation of OR, YES, and AND logic gates using ATP and cocaine as input signals.

A second ATP/cocaine aptamer-based logic gate nano-system [80] has been demonstrated which utilises hexagonal DNA nanostructures [103] that can connect to each other laterally via aptamer locks. Four pairs of aptamer locks were embedded to connect two hexagonal DNA origami structures via side-by-side dimerization and dissociation of the structure was optimised. As a result, the yield for the dimeric form of the structure reached 89% which was decreased to 24% in the presence of 5 mM ATP in 2 h. Similarly, the cocaine aptamer lock was used to mediate dimerization with dimer constituting 87% and 33% of the total in the absence or presence of 5 mM cocaine, respectively. Finally, combining the two aptamer locks formed a DNA origami trimer. Each DNA origami monomer was labelled by 0 to 2 streptavidin flags to distinguish them. The trimer was designed to dissociate into dimer and monomer in the presence of either ATP or cocaine or to dissociate into monomer in the presence of both signals. The DNA origami trimer was produced with 80% yield, which decreased to 19% in the presence of either ATP or cocaine and to 2.8% in the presence of both signals. The dimeric form (approximately 40%) that was observed in the presence of either signal clearly demonstrated controlled dissociation with low crosstalk.

Overall, aptamer locks and split aptamers offer the capability for the dynamic transformation of DNA origami structures that can produce a response as a result of target molecule recognition. The detection of target molecules such as ATP, Na^+^, K^+^, GTP, PDGF, cocaine, and PfLDH have been converted to dynamic transformations of DNA origami structures, as evaluated by AFM, TEM, or optical tweezers as well as agarose gel electrophoresis or tracked real-time by fluorescence signal alteration, derived from FRET or quenchers. As each DNA nanostructure can possess more than one aptamer module, AND gates can be easily constructed by integrating orthogonal aptamer modules. Designing sequential reactions of DNA origami structure interactions allows various kinds of logic gate to be demonstrated. In all cases, high efficiency of transformation control is challenging. Aptamer sequences themselves, along with complementary sequences and the position and multivalency of aptamer modules need to be further optimised for improved performance.

## 4. Aptamers for Cell Targeting

Analogously to their protein antibody counterparts, DNA aptamers have been developed which bind to specific receptors that are displayed on the surface of certain cell types (e.g., cancer cells) [104]. In some cases, aptamers binding to these receptors can promote the uptake of the conjugated structures by the cell as well as triggering cellular signals. This has been utilised in a number of cases to target DNA nanostructures to specific cells with therapeutic goals, as has recently been reviewed by Kim et al., including a useful table of cell selectivity of aptamers [105]. For example, Sgc8c, also used in the programmable DNA origami robot mentioned above [70], targets protein tyrosine kinase 7 (PTK7), a transmembrane receptor that is highly expressed in cancer cell lines including T-cell acute lymphoblastic leukemia [76], while the MUC-1 aptamer, S2.2, which is also used in DNA nanosphere work [88], targets MUC-1 receptor positive cancer cell lines including MCF-7 breast cancer cells [89]. The nucleolin receptor is displayed on rapidly proliferating cells including various cancer cells [92,93] and is an attractive target that is bound by the AS1411 aptamer.

These cell targeting aptamers are widely employed to functionalise DNA nanostructures with examples including an icosahedral DNA nanostructure [106] or ring-shaped DNA nanostructure [107], both of which have been attached to S2.2 to deliver doxorubicin (Dox, dsDNA intercalating anti-cancer drug). Dox has proved to be a popular drug for aptamer modified DNA origami-based delivery: It has been used in a long linear DNA nanostructure decorated with a Sgc8c or an AS1411 motif [108]; a fluorophore labelled dendritic DNA nanostructure with Sgc8c for specific delivery to cancer cells in vitro [109] and DNA tetrahedron with both AS1411 and MUC-1 aptamers delivering Dox [110]. Other examples of targeting origami to cells via aptamers include Y or X shaped DNA nanostructures with Sgc8c, TC01 (aptamer targeting), and Sgc4f [111]; a tetrahedral DNA nanostructure with trivalent AS1411 which reduces the growth of cancer cells [112] along with many others [113,114].

It is noteworthy that nanoparticles in a certain size range (typically 20–200 nm) are passively accumulated to solid cancer tumours by the enhanced permeability and retention (EPR) effect and then are taken in to cells by endocytosis [115], which is encouraging for DNA nanoscience as DNA origami structures are typically within this size range. So far, a 30 helix bundle (HB) DNA origami tube decorated with up to 62 CpG motifs for TLR9 mediated immune response [27]; 6HB tubular or triangular DNA origami structures carrying Dox [116]; a twisted DNA origami tube with tuneable Dox capacity and releasing rate [117] and a 26HB rod-like DNA origami structure carrying daunorubicin [118] have been used to take advantage of this effect. A systematic comparison of cellular uptake efficiency among DNA nanostructures and 3D DNA origami structures with gold nanoparticles (AuNP) [119] have shown in vitro cell uptake of DNA origami structures, while in vivo passive accumulation and cellular intake of various shapes of naked DNA origami structures carrying Dox [120] or gold nanorod (AuNR) [121] as well as lipid membranes and PEG coated octahedral DNA origami structures [122] have been demonstrated.

Passive DNA origami delivery with Dox [116] or AuNRs [121], integrated with MUC-1 aptamer (S2.2) to enhance cell targeting of a triangular DNA origami structure, has been reported where the structure carries both Dox and AuNR as anticancer drugs (Figure 4A) [87]. AuNR synergistically suppressed the expression of P-glycoprotein and the growth of multidrug resistant MCF-7 cells upon NIR irradiation via the hyperthermia effect [123] and the work successfully demonstrated an aptamer derived cell intake enhancement of the DNA origami structure. Further development allowed the attachment of two capped p53 gene modules to the triangular DNA origami carrying Dox, designed to be released and expressed after delivery into the cells, which was tested and showed efficacy in mice [124]. Another report utilised the C2NP aptamer, which recognises CD30 positive cancer cells including K299 and triggers a signalling pathway leading to apoptosis at high concentration [95]. A DNA rectangle decorated with four or 16 such aptamers enhanced cell specific Dox delivery and apoptosis induction [94].

Recently, a DNA origami nanorobot that can target tumours in mice by inhibiting their growth, has been reported (Figure 4B) [91]. In the design, the authors employed AS1411 not only for targeting cancer cells, but also to regulate the mechanical transformation of the DNA nanorobot to expose the cargo at the focus. They first loaded bioactive thrombin-DNA conjugates to four positions of the same surface of a rectangular DNA origami structure by hybridisation. Then, the DNA structure was rolled and “fastened” by six pairs of AS1411 aptamer locks to form a hollow tube. The locks are opened upon interaction with nucleolin. The nanorobot was further decorated with eight AS1411 strands to enhance cell targeting. Thrombin was kept inside the hollow structure to protect it from the innate coagulation system during delivery and was exposed to surroundings only when the nanorobot reached nucleolin positive cells, causing a coagulation cascade that eventually induced necrosis of tumour tissue. Using MDA-MB-231 model mice bearing a human breast cancer tumour as well as C57BL/6J mice injected with B16-F10 melanoma tumour cells, they successfully demonstrated cancer tissue targeting, designed necrosis, tumour growth inhibition, and enhancement of survival time.

## 5. Conclusions and Perspective

In this review, we have summarised the various examples wherein DNA aptamers have been combined with DNA origami and noted that the work can be separated into four distinct areas. In the first, aptamers are used as protein immobilisation modules capable of capturing and arranging proteins in defined patterns and with defined order. This may even allow the control and manipulation of protein to produce designed, protein-based nanomachines, however on a DNA origami framework. The second and the third areas show how aptamers can be used to initiate and control structural changes in DNA origami, allowing extremely sensitive detection and linking the output of molecular logic gates to conformational dynamism. Finally, cell targeting aptamers enable the specificity of drug delivery by DNA origami carriers. As listed in Table 1, the majority of the research relies on a limited number of established aptamers to demonstrate the feasibility of designs while some have utilised DNA origami to expand the applications of newly developed aptamers [57,77,83,85]. There are more aptamers, both well-characterised or novel, that are potentially useful for the functionalisation of DNA origami structures with various potential applications. Some have already been used in DNA nanostructure research or other bioconjugate research reviewed elsewhere [4,12,45,112]. As demonstrated by the work replacing an ATP aptamer lock with a new split aptamer system to enhance the efficiency of DNA origami device transformation [78,83], seeking alternative aptamers could overcome a general problem, which is that the aptamer affinity generally decreases upon attachment to DNA origami. This is likely due to steric and electrostatic effects. An intelligent DNA nanorobot with AS1411 for both cell targeting and drug release [91] exemplifies the new possibility of the development of sophisticated DNA origami devices, utilising multiple aptamers simultaneously to achieve synergistic effects or programmability, and we hope that this brief review encourages the further development of improved aptamers for integration with DNA origami.

## Figures and Tables

**Table 1:** Summary of aptamers utilised in DNA origami research.

Name	Target	Sequences	Length/DNA or RNA	Employed by	Ref.
HD1	Thrombin (exosite I)	GGTTGGTGTGGTTGG	15nt ssDNA	[53,54,59,61]	[3]
HD22	Thrombin (exosite II)	AGTCCGTGGTAGGGCAGGTTGGGGTGACT	29nt ssDNA	[18,56,59,63]	[52]
NU172 ^1^	Thrombin	CGCCTAGGTTGGGTAGGGTGGTGGCG	26nt ssDNA	[56]	[55]
36t	PDGF (platelet derived growth factor)	CACAGGCTACGGCACGTAGAGCATCACCATGATCCTGTGT	40nt ssDNA	[18]	[51]
41t	PDGF	TACTCAGGGCACTGCAAGCAATTGTGGTCCCAATGGGCTGAGTAT	45nt ssDNA	[70,71,72]	[51]
SL12	VEGF (vascular endothelial growth factor)	ATACCAGTCTATTCAATTGGGCCCGTCCGTATGGTGGGTGTGCT ^3^	44nt ssDNA	[72]	[73]
TE17	CCRF-CEM cell	CAGCTACGCAATACAAAACTCCGAACACCTGCTTCTGACTGGGTGCTG	48nt ssDNA	[70]	[74]
sgc8c	PTK7	ATCTAACTGCTGCGCCGCCGGGAAAATACTGTACGGTTAGA	41nt ssDNA	[70]	[75,76]
2008s	PfLDH	CTGGGCGGTAGAACCATAGTGACCCAGCCGTCTAC	35nt ssDNA	[57,77]	[60]
AFB1 aptamer	AFB1	GTTGGGCACGTGTTGTCTCTCTGTGTCTCGTGCCCTTCGCTAGGCCCAC	49nt ssDNA	[58]	[62]
ATP aptamer	ATP	ACCTGGGGGAGTATTGCGGAGGAAGGT ^4^	27nt ssDNA	[78,79,80]	[81,82]
ATP aptamer	ATP	1: CTAcUACCTGGGGGAGTAT ^5^ 2: TGCGGAGGAAGGTcUAG	43nt ssDNA	[83]	[84]
aptakiss and GTP switch	GTP	aptakiss: UGCUCGGCCCCGCGAGCA GTPswitch: UCCGAAGUGGUUGGGCUGGGGCGUGUGAAAACGGAGTPswitch mutant: UCCGAAGUGGUUGGGCUGGGCGUGUGAAAACGGA	18nt RNA/35nt RNA/34nt RNA	[85]	[86]
S2.2	MUC-1	GCAGTTGATCCTTTGGATACCCTGG	25nt ssDNA	[87,88]	[89]
*cocaine aptamer*	*cocaine*	*GGGAGACAAGGATAAATCCTTCAATGAAGTGGGTCTCCC* ^6^	*39nt ssDNA*	[79,80]	[90]
*AS1411* ^2^	*Nucleolin*	*GGTGGTGGTGGTTGTGGTGGTGGTGG*	*26nt ssDNA*	[91]	[92,93]
C2NP	CD30	ACTGGGCGAAACAAGTCTATTGACTATGAGC	32nt ssDNA	[94]	[95]
zif268 binding site	zif268	CTGCGTGGGCGTGTTTTCACGCCCACGCAG	30nt ssDNA	[64,66,69]	[66]
AZP4 binding site	AZP4	CTTACGTGGCATGTTTCATGCCTCGTAAG	29nt ssDNA	[66,69]	[66]
GCN4 binding site	GCN4	CTTCATGAGTCATGCGTTTTCGCATGACTCATGAAG	36nt ssDNA	[64,67]	[67]

^1^ Also known as ARC2172. ^2^ Also known as AGRO100. ^3^ Derivative of VEa5 [96]. ^4^ In [78,80] a single G at 3′ end was added as a result of optimisation. ^5^ Split aptamer. cU in the sequences are dye modified nucleosides. ^6^ Derivative of MNS-4.1 [97]. AFB1: aflatoxin B1.
